# Development and validation of clinical prediction models for cardiorespiratory fitness in atrial fibrillation patients following radiofrequency catheter ablation

**DOI:** 10.3389/fcvm.2025.1659905

**Published:** 2025-08-29

**Authors:** Guiling Zhao, Jian Sun, Qianji Che, Wenqing Xu, Mengmeng Song, Doa El-Ansary, Roger Adams, Jia Han, Shu Meng, Yigang Li

**Affiliations:** ^1^ Department of Cardiology, Shuguang Hospital Affiliated to Shanghai University of Traditional Chinese Medicine, Shanghai, China; ^2^ Department of Cardiology, Xinhua Hospital Affiliated to Shanghai Jiao Tong University School of Medicine, Shanghai, China; ^3^ Department of Sport Rehabilitation, School of Sports and Health, Shanghai University of Sport, Shanghai, China; ^4^ Department of Rehabilitation, Shuguang Hospital Affiliated to Shanghai University of Traditional Chinese Medicine, Shanghai, China; ^5^ School of Health and Biomedical Sciences, Royal Melbourne Institute of Technology University, Melbourne, VIC, Australia; ^6^ Department of Surgery, University of Melbourne, Melbourne, VIC, Australia; ^7^ UC Research Institute for Sport and Exercise, Faculty of Health, University of Canberra, Canberra, ACT, Australia; ^8^ College of Rehabilitation Sciences, Shanghai University of Medicine and Health Sciences, Shanghai, China

**Keywords:** atrial fibrillation, exercise test, oxygen uptake, metabolic, equivalents, regression analysis

## Abstract

**Background:**

Assessment of cardiorespiratory fitness (CRF) is imperative in patients with atrial fibrillation (AF) who have had radiofrequency catheter ablation (RFCA). This study aimed to develop and validate CRF prediction models in this population.

**Methods:**

141 AF patients with RFCA were recruited. The cardiopulmonary exercise test was used to assess CRF with VO_2peak_ and METs_max_. Multidimensional predictors (demographics, serum biomarkers, cardiovascular parameters, and motor function parameters) were analyzed through Spearman correlation analysis and stepwise multivariate linear regression analysis. The internal validity of the prediction equation was tested by paired Student's *t*-test, Pearson correlation analysis and Bland-Altman analysis.

**Results:**

Sex, BMI, ln NT-proBNP, glucose (GLU), 6-minute walking distance (6MWD), and systolic blood pressure (SBP) were found to be significantly associated with CRF in this population. Multivariate linear regression generated the equations: VO_2peak_ = 35.080 − 0.286 * BMI − 1.927 * Sex − 1.090 * ln NT-proBNP + 0.011 * 6MWD − 0.039 * SBP − 0.512 * GLU, and METs_max_ = 9.646 − 0.447 * Sex − 0.260 * ln NT-proBNP − 0.140 * GLU − 0.078 * BMI − 0.016 * SBP + 0.004 * 6MWD, (VO_2peak_: adjusted R^2^ = 0.506, and METs_max_: adjusted R^2^ = 0.469, both *P* < 0.01). Pearson correlations between the predicted values and the measured values showed good validity (VO_2peak_: *r* = 0.616, and METs_max_: *r* = 0.581, both *P* < 0.01). The Bland-Altman analysis showed that the predicted VO_2peak_ values were slightly lower than the measured values (mean difference = −0.13; 95% limits of agreement: −5.20 to 4.93), while the predicted METs_max_ values were in close agreement with the measured values (mean difference = −0.00; 95% limits of agreement: −1.59 to 1.59).

**Conclusion:**

Sex, BMI, NT-proBNP, glucose, 6MWD, and SBP are robust predictors of VO_2peak_ and METs_max_ in AF population after RFCA. This study generates and internal validates the first multivariable CRF prediction models with easy-to use clinical paraments in AF patients after RFCA, thereby providing safe and effective alternatives to conventional CPX, which may help to optimize personalized patient management.

## 1 Introduction

Atrial fibrillation (AF) is one of the most prevalent cardiac arrhythmias with the rising incidence driven by an aging population, which is strongly associated with adverse outcomes such as stroke and heart failure (1, 2). While radiofrequency catheter ablation (RFCA) is listed as a class I recommendation for rhythm control, long-term follow-up studies have demonstrated that the incidence of late arrhythmia recurrence (defined as recurrence occurring more than 12 months post-ablation) can reach up to 30%, highlighting the urgent need for prognostic assessment tools (3–5).

Cardiorespiratory fitness (CRF) is the maximal aerobic capacity quantified by peak oxygen uptake (VO_2peak_) and maximal metabolic equivalents (METs_max_) and has been established as a robust prognostic indicator in cardiovascular diseases (6). Among AF patients, higher CRF is independently associated with reduced risk of arrhythmia recurrence and all-cause mortality after ablation (7, 8). Notably, each 1-metabolic equivalent (MET) increase in CRF correlates with a 20% decrease in AF recurrence risk (9). While cardiopulmonary exercise test (CPX) remains the gold standard for CRF assessment, the implementation of CPX in this population faces three major challenges: First, the prevalence of AF increases with age. Data from the China Health and Retirement Longitudinal Study (CHARLS) show that 7% of Chinese adults aged 60 and above experience frailty (10). Compared to those without AF, individuals with AF are more prone to frailty, falls, and declines in physical function, making it difficult for them to meet the effective testing criteria (Respiratory Exchange Ratio ≥ 1.05) (11). Second, in clinical practice, we have observed that some patients experience kinesiophobia and refuse to undergo maximal exercise testing (12). More importantly, CPX equipment is costly and requires specialized training, limiting its application in primary care settings (13).

To date, safe and effective alternatives for evaluating CRF in AF population after RFCA have remained conspicuously absent (14–16). Emerging evidence suggests that motor function assessments, including sit-to-stand tests and 6-minute walk test, have a close relationship with CRF in cardiovascular population (17, 18). Therefore, this study aims to develop and validate CRF assessment predictive models in AF patients after RFCA using accessible clinical indicators, including demographic information, serum biomarkers, cardiovascular parameters, and motor function parameters, so as to address a critical CRF prediction gap in this population.

## 2 Materials and methods

### 2.1 Ethical approval

This study was conducted in accordance with the 1975 Declaration of Helsinki. It was approved by the Medical Ethics Committee of Xinhua Hospital Affiliated to Shanghai Jiao Tong University School of Medicine (Approval Number: XHEC-C-2024-190-1) and registered at the Chinese Clinical Trial Registry (Registration ID: ChiCTR2400094326; URL: http://www.chictr.org.cn). All patients provided written informed consent for study participation.

### 2.2 Sample size calculation

According to the preliminary experiment, we used G*power 3.1 software (Heinrich Heine University Düsseldorf, Germany) to estimate the required sample size. The analysis was conducted with the following parameters: the effect size *f*^2^ = 0.15, 1 − *β* = 80%, *α* = 0.05, and the number of predicted variables was 5–7. Finally, 92–103 participants were needed.

### 2.3 Study population

This prospective observational cohort study initially enrolled 145 AF patients who underwent RFCA at Xinhua Hospital affiliated to Shanghai Jiao Tong University School of Medicine, between May 2024 and February 2025.

All AF patients were diagnosed via standard 12-lead ECG or 24-hour Holter monitoring, and classified according to the 2023 ACC/AHA/ACCP/HRS guidelines (paroxysmal AF: self-terminating within 48 h; persistent AF: sustained > 7 days or requiring cardioversion; long-standing persistent AF: Continuous > 12 months) (1). Antiarrhythmic drugs (e.g., amiodarone) were discontinued for ≥ 5 half-lives pre-procedure. Within 48 h before the procedure, transesophageal echocardiography was performed to exclude intracardiac thrombus, supplemented by cardiac computed tomography angiography when clinically feasible. All procedures were performed under local anesthesia and guided by the CARTO3 navigation system (Biosense Webster, Inc., Irvine, USA). The THERMOCOOL SMARTTOUCH SF catheter was used as its 56-hole tip irrigation facilitating cooling at low flow rate, thus easing the fluid management process. Pulmonary vein isolation (PVI) was performed in all patients. Additional ablation including left atrial roof line, anterior septal, posterior and inferior lines, mitral isthmus (MI) and cavo-tricuspid isthmus (CTI) lines, complex fractionated electrograms (CFAE) modification, and ablation of ganglionated plexi and extra-PV triggers, were performed when deemed necessary. For patients not achieving sinus rhythm post-ablation, low-energy (≤15 J) intracardiac cardioversion was delivered via catheters positioned in the right atrium and coronary sinus/left atrium. All operations were performed by experienced physicians (>50 annual cases).

Inclusion criteria were: AF patients aged between 40 and 80 years old who underwent RFCA in the last 3–12 months (3), documented sinus rhythm with a heart rate ranging from 60 to 100 beats per minute, and with written informed consent given. Exclusion criteria included: patients with contraindications to CPX as defined by the American Heart Association, significant musculoskeletal system diseases (e.g., fractures, serious soft tissue injuries) or severe chronic diseases (e.g., cerebrovascular, pulmonary, hepatic, or renal impairment), patients with cognitive dysfunction, and those who had participated in other intervention trials within the past 90 days. After excluding atrial flutter (*n* = 1), non-consent (*n* = 1), and severe respiratory comorbidities (*n* = 2), data from 141 participants were analyzed (Supplementary Figure S1).

### 2.4 Data collection

The retrospective data of all patients included: (1) Demographic and anthropometric data: Age, gender, height, weight, and waist circumference, body mass index (BMI), blood pressure, smoking history, alcohol consumption, medical history, and current medications were recorded. The BMI was calculated as weight (kg) divided by height squared (m^2^). (2) Laboratory data: Fasting venous blood samples were collected in the morning to measure *N*-terminal pro-B-type natriuretic peptide (NT-proBNP), hemoglobin (HGB), glucose (GLU), serum creatinine (Cr), low-density lipoprotein cholesterol (LDL-C), high-density lipoprotein cholesterol (HDL-C), total cholesterol (TC), triglycerides (TG) and estimated glomerular filtration rate (eGFR) levels. (3) Cardiac function data: Two-dimensional transthoracic echocardiography was used to measure left ventricular end-diastolic dimension (LVEDD), left atrial anteroposterior diameter (LAD), left ventricular ejection fraction (LVEF) and systolic pulmonary artery pressure (PAP).

### 2.5 Assessment of cardiorespiratory fitness

Cardiopulmonary exercise test was conducted on an electronically braked cycle ergometer according to *American Heart Association guidelines* (13). The baseline phase included 3 min of seated rest. The warm-up phase consisted of 3 min of unloaded cycling (55–65 rpm). In the incremental phase, the workload was continuously increased at a constant rate of 10–15 watts per minute (ramp protocol) until the patient experienced voluntary fatigue or met the termination criteria. The recovery phase was 3 min of unloaded cycling (30 rpm). Throughout the process, output from a continuous 12-lead electrocardiogram and pulse oximetry were monitored, and blood pressure was measured every 3 min. VO_2peak_ was directly measured using the Quark PFT4 Ergo metabolic cart (COSMED, Italy) and defined as the highest 30 s average value during maximal effort, normalized to body weight (ml·kg^−1^·min^−1^). METs_max_ was calculated as VO_2peak_ divided by 3.5 ml·kg^−1^·min^−1^ (13).

### 2.6 Assessment of motor function

(1) Time up-and-go test (TUG): Participants sat on a 46 cm high chair with back against the chair, arms resting on the chair's arms. Upon receiving the “Go” command, they stood up and walked at a comfortable and safe pace to a line on the floor 3 m away, turned, returned to the chair, and sat down again. The total completion time was recorded in seconds (19). (2) Five-times sit-to-stand test (FTSTS): Participants performed five consecutive sit-to-stand cycles from a 46 cm high chair placed against a wall. The initial position required the ankles to be in a neutral alignment, with feet flat and the arms folded across the chest. Upon receiving the “Go” command, participants were instructed to fully extend their knees and hips during the standing phase and ensure complete contact with the chair during the sitting phase (20). The total time to complete five cycles was recorded. (3) 6-minute walk distance (6MWD): The 6MWD was performed according to the American Thoracic Society guidelines on a 30 m indoor walkway with colored cones marking turn-around points (21). A certified cardiac rehabilitation therapist assessed the baseline heart rate and blood pressure, and gave standardized instructions. Participant then walked for six min at their self-selected maximal pace. Post-test measurements, including 6MWD, heart rate and blood pressure during the recovery phase were recorded.

All participants abstained from caffeine for ≥12 h and fasted for ≥3 h before the tests, and wore comfortable clothing and shoes. To minimize the interference of fatigue, all motor function tests were completed within one week. TUG and FTSTS were performed on the same day with 5 min seated recovery interval between them, whereas the 6MWD commenced precisely 30 min after the completion of TUG/FTSTS. CPX was conducted on a separate day.

### 2.7 Statistical analysis

All statistical analyses were conducted using SPSS 25.0 (IBM Ink., Armonk, NY) under the supervision of a medical statistician expert. Normally distributed continuous variables were analyzed using Student's *t*-test, reported as mean ± SD. Non-normally distributed continuous variables were compared via the Mann–Whitney *U* test with median (Q1, Q3) presentation. Categorical variables were analyzed by the Chi—square (*χ*^2^) test, and the results were presented as percentages (%). Participants were randomly allocated into a derivation cohort (*n* = 105) and a validation cohort (*n* = 36) at a ratio of 3:1. Variables demonstrating statistical trends (*P* < 0.1) in the correlation analysis with VO_2peak_ and METs_max_ were initially selected as candidate independent variables. Stepwise regression (forward entry *α* ≤ 0.05, backward retention *α* ≥ 0.1) was employed to develop the predictive equations. The robustness of the models was evaluated through multiple means. Normality was assessed using histograms of standardized residuals. Multicollinearity was examined by calculating the variance inflation factor (VIF), with a threshold of VIF < 5. Residual independence was evaluated using the Durbin—Watson statistic, with an acceptable range of 1.5–2.5. The goodness-of-fit of the models was determined by *F*-tests from ANOVA, and reported as adjusted R^2^. For internal validation, predicted and measured values in the validation cohort were compared using Pearson correlation coefficients and paired Student's *t*-test, with Bland-Altman agreement analysis to evaluate accuracy and systematic bias. For all analyses, *P* < 0.05 indicated statistically significant.

## 3 Results

### 3.1 Baseline characteristics

This study enrolled 141 AF patients who underwent RFCA, with detailed characteristics presented in Table 1. The average age was 68.7 ± 7.58 years, including 62.4% males and 56.7% persistent AF patients. Comorbidities included hypertension (96 patients), diabetes (27 patients), CAD (83 patients), and prior stroke (31 patients). Among these patients, 54.6% received ACEIs/ARBs/ARNI therapy, 81.6% NOACs, 86.5% amiodarone, 53.2% β-blockers, 23.4% antiplatelet agents, and 68.1% statins. Echocardiographic parameters demonstrated a mean LAD of 38.98 ± 4.96 mm, LVEED of 47.6 ± 4.61 mm, and LVEF of 65.72 ± 5.21%. Motor function tests revealed FTSTS (13.85 ± 4.08 s), TUG (9.35 ± 2.81 s), and 6MWD (397.0 8 ± 60.4 m). CPX results indicated VO_2peak_ and METs_max_ were 16.29 ± 3.20 ml·kg^−1^·min^−1^ and 4.63 ± 0.98 ml·kg^−1^·min^−1^, respectively. No significant differences existed in demographic, blood biochemistry, motor function, and cardiopulmonary function parameters between validation and derivation cohorts (*P* > 0.05).

**Table 1:** Descriptive characteristics and cardiopulmonary exercise test variables of study participants.

Indicator	Total *N* = 141	Derivation cohort *N* = 105	Validation cohort *N* = 36	*t*/*χ*^2^/*Z*	*P* value
Age (years)	68.7 ± 7.58	68.78 ± 7.47	68.47 ± 8.02	0.21	0.834
Male, *n* (%)	88 (62.4)	65 (61.9)	23 (63.9)	0.045	0.832
BMI (kg/m^2^)	24.74 ± 3.49	24.78 ± 3.42	24.65 ± 3.75	0.188	0.851
Height (m)	1.67 ± 0.08	1.67 ± 0.08	1.67 ± 0.07	−0.075	0.94
Weight (kg)	69.53 ± 12.25	69.56 ± 11.99	69.46 ± 13.16	0.04	0.968
SBP (mmHg)	121.53 ± 20.42	123.33 ± 16.47	116.28 ± 28.70	1.397	0.165
DBP (mmHg)	77.57 ± 10.03	78.11 ± 10.09	79.37 ± 16.17	−0.433	0.667
Current smoking, *n* (%)	16 (11.3)	9 (56.3)	7 (43.8)	2.162	0.141
Alcohol consumption, *n* (%)	24 (17.0)	18 (75.0)	6 (25.0)	0.004	0.948
Persistent/long-standing persistent AF, *n* (%)	80 (56.7)	62 (77.5)	18 (22.5)	0.894	0.344
Paroxysmal AF, *n* (%)	61 (43.3)	43 (70.5)	18 (29.5)	0.894	0.344
CHA_2_DS_2_-VASc	4.50 ± 1.64	4.51 ± 1.63	4.44 ± 1.70	0.220	0.827
Disease					
Hypertension, *n* (%)	96 (68.1)	74 (77.1)	22 (22.9)	1.082	0.298
Diabetes, *n* (%)	27 (19.1)	21 (77.8)	6 (22.2)	0.192	0.661
CAD, *n* (%)	83 (58.9)	62 (74.7)	21 (25.3)	0.006	0.940
stroke/TIA, *n* (%)	31 (22.0)	22 (71.0)	9 (29.0)	0.256	0.613
Medication					
ACEIs/ARBs/ARNI, *n* (%)	77 (54.6)	61 (79.2)	16 (20.8)	2.015	0.156
NOACs, *n* (%)	115 (81.6)	84 (73.0)	31 (27.0)	0.666	0.415
Amiodarone, *n* (%)	122 (86.5)	92 (75.4)	30 (24.6)	0.135	0.714
*β*-blockers, *n* (%)	75 (53.2)	59 (78.7)	16 (21.3)	1.486	0.223
Antiplatelet agents, *n* (%)	33 (23.4)	25 (75.8)	8 (24.2)	0.038	0.846
Statins, *n* (%)	96 (68.1)	70 (72.9)	26 (27.1)	0.381	0.537
Cardiac function					
LVEED (mm)	47.6 ± 4.61	48.02 ± 4.36	46.34 ± 5.16	1.884	0.062
LAD (mm)	38.98 ± 4.96	39.38 ± 4.92	37.79 ± 4.98	1.644	0.103
LVEF (%)	65.72 ± 5.21	65.74 ± 5.23	65.65 ± 5.24	0.085	0.932
PAP (mmHg)	39.14 ± 13.19	41.11 ± 13.95	33.42 ± 8.53	3.906	<0.001
Laboratory data					
NT-proBNP (pg/ml)	374.15 (86.49–265.09)	438.41 (85.63–288.59)	186.69 (87.83–227.22)	−1.227	0.220
HGB (g/L)	139.07 ± 14.71	137.8 ± 15.43	142.69 ± 11.85	−1.723	0.087
GLU (mmol/L)	5.94 ± 1.18	5.89 ± 1.17	6.07 ± 1.23	−0.791	0.431
Cr (umol/L)	84.67 ± 100.54	88.65 ± 11.08	73.04 ± 14.26	0.803	0.423
TC (mmol/L)	3.86 ± 0.92	3.85 ± 0.96	3.91 ± 0.83	−0.345	0.731
TG (mmol/L)	1.16 ± 0.60	1.19 ± 0.60	1.07 ± 0.58	1.077	0.283
HDL-C (mmol/L)	1.25 ± 0.31	1.23 ± 0.29	1.32 ± 0.36	−1.585	0.115
LDL-C (mmol/L)	2.22 ± 0.86	2.23 ± 0.89	2.18 ± 0.76	0.284	0.777
eGFR (ml/min)	85.53 ± 24.97	84.4 ± 26.15	88.83 ± 21.14	−0.918	0.36
Motor function					
FTSTS (s)	13.85 ± 4.08	13.85 ± 4.29	13.86 ± 3.43	−0.021	0.984
TUG (s)	9.35 ± 2.81	9.37 ± 3.00	9.30 ± 2.18	0.125	0.901
6MWD (m)	397.08 ± 60.4	394.64 ± 62.31	404.2 ± 54.65	−0.819	0.414
Cardiorespiratory fitness					
VO_2peak_ (ml·kg^−1^·min^−1^)	16.29 ± 3.20	16.09 ± 3.18	16.88 ± 3.22	−1.294	0.198
METs_max_ (ml·kg^−1^·min^−1^)	4.63 ± 0.98	4.57 ± 0.99	4.80 ± 0.96	−1.232	0.22
%predicted VO_2peak_	71.4 ± 12.59	70.77 ± 12.63	73.25 ± 12.47	−1.019	0.31
Power (Watt)	81.05 ± 26.37	80.72 ± 25.76	82.00 ± 28.44	−0.25	0.803
RER	1.17 ± 0.12	1.18 ± 0.13	1.14 ± 0.08	1.99	0.05
VE/VCO_2_ slope	28.43 ± 4.26	28.35 ± 4.35	28.68 ± 4.06	−0.421	0.675
HR_rest_ (bpm)	74.72 ± 13.9	73.60 ± 13.86	78.00 ± 13.68	−1.649	0.101
HR_max_ (bpm)	107.68 ± 16.59	106.46 ± 16.83	111.25 ± 15.54	−1.503	0.135

6MWD, 6-minute walk distance; ACEIs/ARBs/ARNI, angiotensin-converting enzyme inhibitors/angiotensin receptor blockers/angiotensin receptor-neprilysin inhibitors; BMI, body mass index; CAD, coronary artery disease; Cr, creatinine; eGFR, estimated glomerular filtration rate; FTSTS, five-times sit-to-stand test; GLU, glucose; HDL-C, high-density lipoprotein cholesterol; HGB, hemoglobin; LAD, left atrial diameter; LDL-C, low-density lipoprotein cholesterol; LVEDD, left ventricular end-diastolic diameter; LVEF, left ventricular ejection fraction; METs_max_, peak metabolic equivalents; NOACs, non-vitamin K antagonist oral anticoagulants; NT-proBNP, N-terminal pro-B-type natriuretic peptide; PAP, pulmonary artery pressure; RER, respiratory exchange ratio; SBP, systolic blood pressure; TC, total cholesterol; TG, triglycerides; TIA, transient ischemic attack; TUG, time up-and-go test; VE/VCO2 slope, minute ventilation/carbon dioxide production slope; VO_2peak_, peak oxygen uptake.

### 3.2 Correlation analysis

As summarized in Table 2, VO_2peak_ in AF patients was significantly correlated with multiple indicators including age (*r* = −0.296, *P* = 0.002), height (*r* = 0.301, *P* = 0.002), BMI (*r* = −0.269, *P* = 0.005), gender (*r* = −0.451, *P* < 0.001), LAD (*r* = −0.306, *P* = 0.002), NT-proBNP (*r* = −0.379, *P* < 0.001), hemoglobin (*r* = 0.316, *P* = 0.001), eGFR (*r* = 0.309, *P* = 0.001), FTSTS (r = −0.303, *P* = 0.002), TUG (*r* = −0.253, *P* = 0.009), 6MWD (*r* = 0.388, *P* < 0.001), and systolic blood pressure (*r* = −0.326, *P* = 0.001). METs_max_ also showed significant correlations with multiple indicators including age (*r* = −0.305, *P* = 0.002), height (*r* = 0.229, *P* = 0.002), BMI (r = −0.272, *P* = 0.005), gender (*r* = −0.448, *P* < 0.001), LAD (*r* = −0.301, *P* = 0.002), NT-proBNP (*r* = −0.369, *P* < 0.001), hemoglobin (*r* = 0.314, *P* = 0.001), eGFR (*r* = 0.302, *P* = 0.002), FTSTS (*r* = −0.308, *P* = 0.001), TUG (*r* = −0.263, *P* = 0.007), 6MWD (*r* = 0.4, *P* < 0.001), and systolic blood pressure (*r* = −0.332, *P* = 0.001). Other indicators such as weight, LVEF, and lipid levels showed no significant correlation with VO_2peak_ and METs_max_.

**Table 2:** Correlations between the study variables and VO_2peak_ and METs_max_.

Study variables	VO_2peak_		METs_max_	
	Correlation coefficient	*P* value	Correlation coefficient	*P* value
Age (year)	−0.296	0.002	−0.305	0.002
Height (m)	0.301	0.002	0.299	0.002
Weight (kg)	−0.004	0.968	−0.008	0.935
BMI (kg/m^2^)	−0.269	0.005	−0.272	0.005
Sex	−0.451	<0.001	−0.448	<0.001
LVEED (mm)	−0.113	0.249	−0.102	0.299
LAD (mm)	−0.306	0.002	−0.301	0.002
LVEF (%)	−0.096	0.330	−0.096	0.332
PAP (mmHg)	−0.185	0.060	−0.180	0.067
NT-proBNP (pg/ml)	−0.379	<0.001	−0.369	<0.001
HGB (g/L)	0.316	0.001	0.314	0.001
GLU (mmol/L)	−0.169	0.085	−0.169	0.086
Cr (umol/L)	0.091	0.356	0.096	0.328
TC (mmol/L)	0.058	0.556	0.064	0.516
TG (mmol/L)	0.072	0.463	0.079	0.422
HDL-C (mmol/L)	−0.127	0.198	−0.123	0.211
LDL-C (mmol/L)	0.122	0.214	0.125	0.203
eGFR (ml/min)	0.309	0.001	0.302	0.002
FTSTS (s)	−0.303	0.002	−0.308	0.001
TUG (s)	−0.253	0.009	−0.263	0.007
6MWD (m)	0.388	<0.001	0.400	<0.001
SBP (mmHg)	−0.326	0.001	−0.332	0.001

6MWD, 6-minute walk distance; BMI, body mass index; Cr, creatinine; eGFR, estimated glomerular filtration rate; FTSTS, five times sit-to-stand test; GLU, glucose; HDL-C, high-density lipoprotein cholesterol; HGB, hemoglobin; LAD, left atrial diameter; LDL-C, low-density lipoprotein cholesterol; LVEDD, left ventricular end-diastolic diameter; LVEF, left ventricular ejection fraction; NT-proBNP, N-terminal pro-B-type natriuretic peptide; PAP, pulmonary artery pressure; SBP, systolic blood pressure; TC, total cholesterol; TG, triglycerides; TUG, time up-and-go test.

### 3.3 VO_2peak_ prediction equation

The final regression equation was: VO_2peak_ (ml·kg^−1^·min^−1^) = 35.080 − (0.286 * BMI [kg/m^2^]) − (1.927 * Sex [male = 0; female = 1]) − (1.090 * ln NT-proBNP [pg/ml]) + (0.011 * 6MWD [m]) − (0.039 * SBP [mmHg]) − (0.512 * GLU [mmol/L]).

Standard error of estimate (SEE) = 2.236 ml·kg^−1^·min^−1^, *R* = 0.731, adjusted *R*^2^ = 0.506.

As shown in Tables 3, 4, all variables in the multiple linear regression model were significantly associated with VO_2peak_ (*P* < 0.05), with variable influence ranked as: NT-proBNP > BMI > Sex > 6MWD > SBP > GLU.

**Table 3:** Standardized and unstandardized coefficients from multiple linear regression analysis to predict VO_2peak_ and METs_max_ in the derivation cohort.

		Unstandardized coefficient	Standardized coefficient	*t*	*P* value	Tolerance	VIF
VO_2peak_ model	(Constant)	35.080		9.796	<0.001
	lnNT-proBNP	−1.090	−0.332	−4.400	<0.001	0.836	1.196
	BMI (kg/m^2^)	−0.286	−0.308	−4.262	<0.001	0.912	1.096
	Sex	−1.927	−0.296	−3.888	<0.001	0.822	1.217
	6MWD (m)	0.011	0.213	2.778	0.007	0.807	1.239
	SBP (mmHg)	−0.039	−0.200	−2.827	0.006	0.954	1.049
	GLU (mmol/L)	−0.512	−0.188	−2.686	0.008	0.973	1.028
METs_max_ model	(Constant)	9.646		8.380	<0.001		
	6MWD (m)	0.004	0.277	3.479	0.001	0.807	1.239
	SBP (mmHg)	−0.016	−0.274	−3.747	<0.001	0.954	1.049
	BMI (kg/m^2^)	−0.078	−0.269	−3.596	0.001	0.912	1.096
	lnNT-proBNP	−0.260	−0.255	−3.268	0.001	0.836	1.196
	Sex	−0.447	−0.221	−2.805	0.006	0.821	1.217
	GLU (mmol/L)	−0.140	−0.166	−2.291	0.024	0.973	1.028

6MWD, 6-minute walk distance; BMI, body mass index; GLU, glucose; lnNT-proBNP, natural log-transformed N-terminal pro-B-type natriuretic peptide; SBP, systolic blood pressure; VIF, variance inflation factor.

**Table 4:** Summaries of multiple linear regression model for predict VO_2peak_ and METs_max_.

Model	*R* ^2^	Adjusted *R*^2^	SEE	*F*	*P* value	Durbin-watson
VO_2peak_ model	0.534	0.506	2.236	18.738	<0.001	1.862
METs_max_ model	0.499	0.469	0.719	16.3	<0.001	2.082

**Figure 1:**
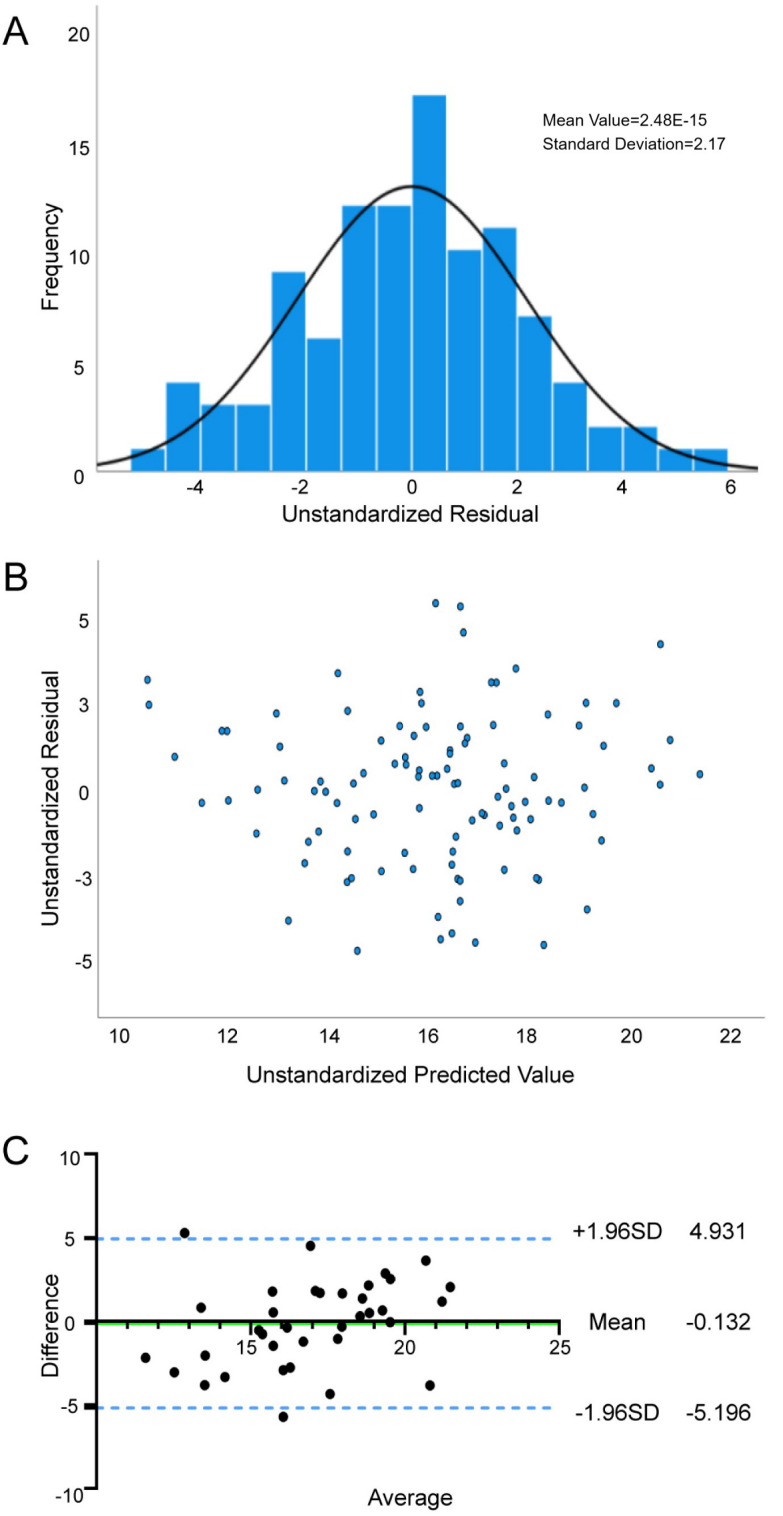
Reliability and validity tests of VO_2peak_ prediction model. **(A)** Normality assessment of residuals in the VO_2peak_ prediction model; **(B)** Residual plot and distribution for the VO_2peak_ regression model; **(C)** Agreement between the measured VO_2peak_ and estimated VO_2peak_ by Bland—Altman difference plot.

The tolerance of the VO_2peak_ prediction model was between 0.807 and 0.973, and the VIF ranged from 1.028 to 1.239, confirming that the variables were independent of each other. The model demonstrated satisfactory goodness-of-fit, with adjusted *R*^2^ of 0.506, SEE of 2.236 ml·kg^−1^·min^−1^, and significant *F*-statistic (*F* = 18.738, *P* < 0.001). The histogram of residuals (Figure 1A) combined with the Kolmogorov–Smirnov test (*P* = 0.20) supported that the residuals were normally distributed. Scatterplot analysis (Figure 1B) revealed that residual dispersion remained relatively constant across the prediction range, which confirmed homogeneity of variance. The Durbin-Watson statistic (1.862) indicated that the residuals of this regression equation were independent of each other.

Internal validation analyses (Table 5) demonstrated strong agreement between predicted and measured VO_2peak_. Pearson correlation analysis was significant (*r* = 0.616, *P* < 0.01), with no systematic bias detected via paired Student's *t*-test (*P* > 0.05). The Bland-Altman analysis (Figure 1C) showed a mean bias of −0.13 (95% LoA: −5.20 to 4.93) between the two values, indicating that the predicted value was slightly lower than the measured value, but the difference was within acceptable limits. These results initially validate the robustness of the VO_2peak_ prediction model integrating NT-proBNP, BMI, Sex, 6MWD, SBP, and GLU.

**Table 5:** Comparison of the measured value and predicted value using paired student's *t*-test and Pearson correlation analysis in the validation cohort.

Model	Mean ± SD	*t*	*P* value	Difference value (95%CI)	*r*
Measured VO_2peak_	16.88 ± 3.22	0.31	0.76	−0.13 (−1.01, −0.27)	0.616
Predicted VO_2peak_	17.02 ± 3.22				
Measured METs_max_	4.8 ± 0.96	−0.00	1.00	0 (−0.27,0.27)	0.581
Predicted METs_max_	4.8 ± 0.76				

### 3.4 METs_max_ prediction equation

The final regression equation was: METs_max_ (ml·kg^−1^·min^−1^) = 9.646 − (0.447 × Sex [male = 0; female = 1]) − (0.260 × ln NT-proBNP [pg/ml]) − (0.140 × GLU [mmol/L]) − (0.078 × BMI [kg/m^2^]) − (0.016 × SBP [mmHg]) + (0.004 × 6MWD [m]).

SEE = 0.719 ml·kg^−1^·min^−1^, *R* = 0.719, adjusted *R*^2^ = 0.469.

Table 1 shows that all the variables included in the regression model were significantly associated with METs_max_ (*P* < 0.05) with variable influence ranked as: 6MWD > SBP > BMI > NT-proBNP > Sex > GLU.

**Figure 2:**
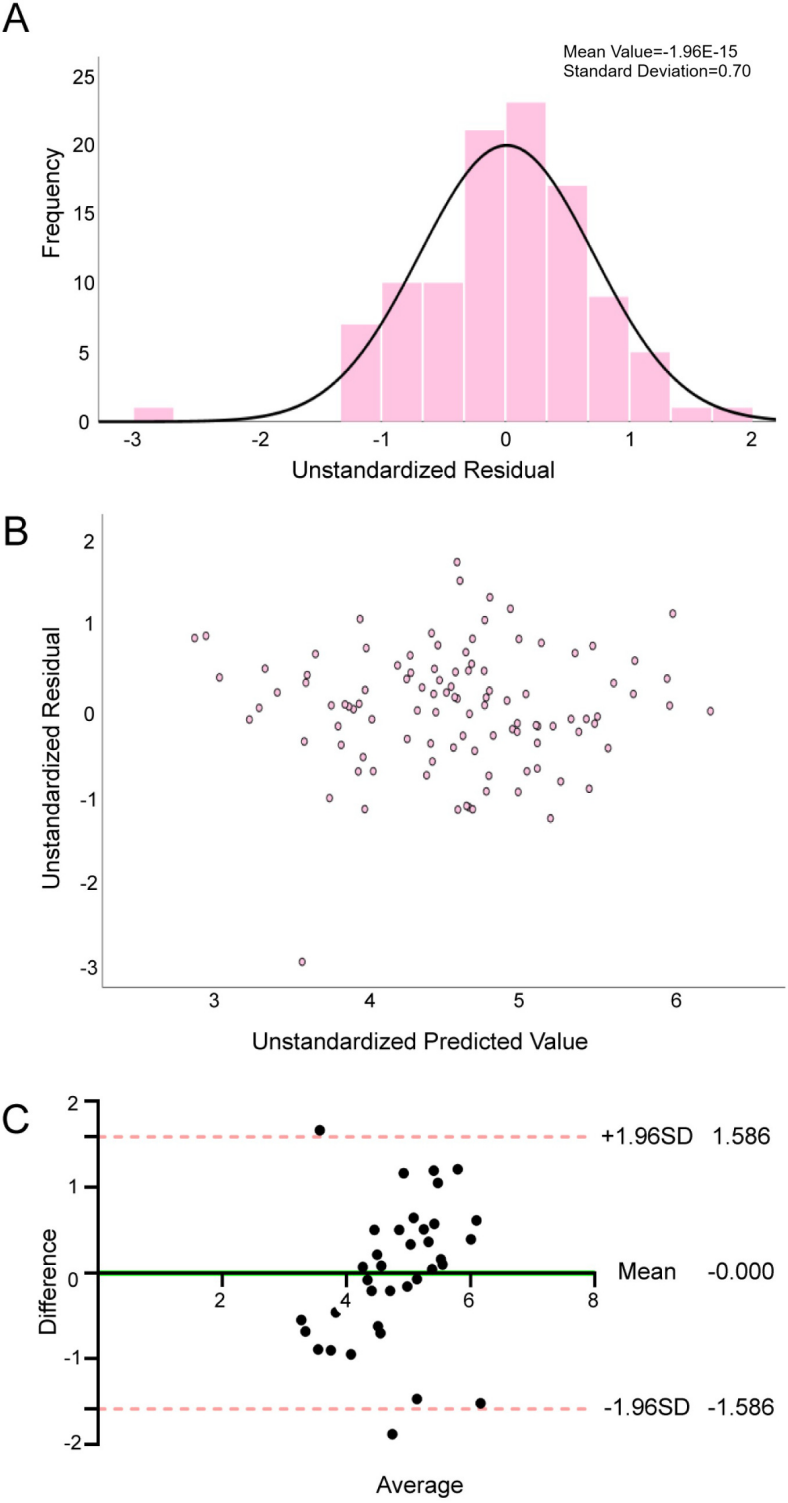
Reliability and validity tests of METs_max_ prediction model. **(A)** Normality assessment of residuals in the METs_max_ prediction model; **(B)** Residual plot and distribution for the METs_max_ regression model; **(C)** Agreement between the measured METs_max_ and estimated METs_max_ by Bland—Altman difference plot.

Tables 3, 4 show the results of the multiple linear regression and the reliability test of the METs_max_ prediction model, respectively. The METs_max_ prediction model demonstrated acceptable multicollinearity metrics (tolerance > 0.1, VIF < 5), confirming variable independence. The regression exhibited satisfactory goodness-of-fit, with adjusted R^2^ of 0.506, SEE of 2.236 ml·kg^−1^·min^−1^, and significant *F*-statistic (*F* = 16.3, *P* < 0.01). Residual diagnostics (Figures 2A,B) supported adherence to regression assumptions: normality was confirmed by Kolmogorov–Smirnov test (*P* = 0.2), homoscedasticity by the residual scatter-plot, and residual independence by the Durbin-Watson statistic (2.082).

Internal validation analyses (Table 5) revealed strong concordance between predicted and measured METs_max_. Pearson correlation analysis was significant (*r* = 0.581, *P* < 0.01), with no systematic bias detected via paired Student's *t*-test (*P* > 0.05). The Bland-Altman analysis (Figure 2C) demonstrated excellent agreement between predicted and measured values, with a negligible mean bias of −0.00 (95% LoA: −1.59 to 1.59), confirming high concordance between the two values. These results initially validate the robustness of the METs_max_ prediction model incorporating 6MWD, SBP, BMI, NT-proBNP, Sex, and GLU.

## 4 Discussion

This study demonstrated that Sex, BMI, 6MWD, SBP, NT-proBNP, and glucose are robust predictors of VO_2peak_ and METs_max_ in a cohort of AF patients following RFCA. The novel predictive model demonstrated superior performance compared to Peterman's equation, it achieved a 53% reduction in the standard error of estimate (2.24 vs. 4.75) and demonstrated enhanced explanatory power (adjusted *R*^2^ = 0.506 vs. 0.43) (16). This improvement holds clinical relevance given the critical role of VO_2peak_ and METs_max_ in quantifying CRF through oxygen utilization and metabolic equivalents (13).

The 6MWD represents a rapid, safe and cost-effective measure that is closely associated with an individual's CRF and is often used to assess the risk of mortality and rehospitalization in heart failure patients (17). Prior intervention studies have demonstrated that a six-week cardiac rehabilitation program can significantly enhance 6MWD and CRF metrics in patients with coronary artery disease, and this study found that 6MWD was closely associated with VO_2peak_ and METs_max_ in AF patients after RFCA, suggesting similar rehabilitation potential in this population (18, 22). These findings are further supported by recent Cochrane reviews, which confirm that exercise interventions can enhance VO_2peak_, reduce AF recurrence, and alleviate AF-related symptoms (23, 24).

The results of the present study showed that male AF patients demonstrated significantly higher VO_2peak_ and METs_max_ compared to females (17.19 ± 3.09 vs. 14.30 ± 2.45 ml·kg^−1^·min^−1^; 4.87 ± 1.02 vs. 4.09 ± 0.7 METs). This gender disparity aligns with previous reports and may be mediated by factors such as cardiac chamber dimensions, cardiac output, and hemoglobin concentrations (25–27).

Multivariable analysis revealed inverse associations of BMI and SBP with CRF, which is consistent with the findings from prior studies (14, 28–31). Excessive epicardial adipose tissue deposition may constrain ventricular diastolic compliance, thereby reducing stroke volume (32, 33). The coexistence of hypertension may further reduce cardiac output through the activation of the renin-angiotensin-aldosterone system and the sympathetic nervous system (34–36). These findings emphasis the potential cardiovascular benefits of systematic weight and blood pressure management in this patient population.

Notably, this study identified fasting glucose as a novel metabolic factor contributing to CRF impairment. Hyperglycemia may exacerbate myocardial fibrosis and left ventricular diastolic dysfunction by activating advanced glycation end products-receptor (AGEs-RAGE) through oxidative stress and inflammation (37–39). This hypothesis is supported by trials showing improvement in CRF with intensive glycemic control (28).

Of particular interest are our findings regarding NT-proBNP. Considering the non-normal distribution of NT-proBNP levels (Shapiro–Wilk *P* < 0.01), natural log-transformation (ln NT-proBNP) was performed before analysis. Multivariable stepwise regression, adjusted for other variables, revealed persistent inverse associations between ln NT-proBNP and CRF: VO_2peak_ (*β* = −0.332, *P <* 0.01) and METs_max_ (*β* = −0.255, *P* < 0.01). The observed relationships likely reflect the biomarker's association with increased ventricular wall stress, myocardial fibrosis, and left ventricular diastolic dysfunction, collectively resulting to a reduction in cardiac output and oxygen delivery capacity during exercise (36, 40, 41). These results gain additional significance considering emerging evidence linking elevated NT-proBNP levels to adverse clinical outcomes and arrhythmia recurrence in heart failure population (42, 43). These findings suggest a dual role for NT-proBNP as both a biomarker of CRF impairment and a potential therapeutic target for functional recovery in AF patients after RFCA.

Finally, our analysis of age-related effects warrants discussion. While the univariate analysis in this study revealed a significant negative correlation between age and both VO_2peak_ and METs_max_, consistent with ATS/ACCP declaration data and previous large-scale cohort studies, confirming age as a crucial factor influencing cardiopulmonary function (44, 45). However, this relationship became nonsignificant after adjusting for sex, BMI, NT-proBNP, glucose, 6MWD, and SBP. This may be attributed to the relatively concentrated age distribution (92.4% aged 60–80 years) and limited sample size. Mediation analysis further revealed that the effect of age on CRF may be primarily mediated through 6MWD (*r* = −0.376, *P* < 0.001) and lnNTproBNP (*r* = 0.401, *P* < 0.001), which is also consistent with the relevant literature (46–49). We acknowledge the potential for collider bias in these analyses and emphasize that these findings represent statistical associations rather than causal inferences.

In summary, this prediction model holds significant clinical value with three key implications: First, the VO_2peak_ and METs_max_ prediction equations incorporating six routinely available clinical parameters (sex, BMI, NT-proBNP, glucose, 6MWD, and SBP) enable rapid outpatient assessment of CRF in AF patients following RFCA, which will provide objective data to guide clinical decision-making. Second, the model can be integrated into electronic health record systems for automated calculations and dynamic monitoring of cardiopulmonary function changes (e.g., quarterly reassessment combining 6MWD and NT-proBNP), potentially enhancing long-term follow-up efficiency. Third, for regions with limited medical resources, this tool may serve as a practical alternative to complex CPX testing. To enhance clinical translation, future directions could include multicenter validation studies to systematically evaluate model performance across diverse clinical scenarios, thereby providing quantitative evidence for developing personalized cardiac rehabilitation protocols. Additionally, prospective cohort studies should be conducted to analyze dynamic changes between CRF and both quality of life and major adverse cardiovascular events (including AF recurrence and all-cause mortality) following RFCA.

## 5 Limitations

We acknowledge several important limitations in our study. First, as a single-center cross-sectional study with a relatively small sample size (*n* = 141), it limited generalizability to broader clinical populations. Second, while we employed stepwise regression—a widely used approach in exploratory clinical research—we recognize its potential limitations in variable selection. Third, external validation has not yet been performed, so the model's performance in heterogeneous populations remains to be verified. Fourth, while our use of conventional echocardiographic parameters (e.g., LAD) rather than more advanced measures like left atrial volume index (LAVi) or strain imaging enhances clinical accessibility, this pragmatic approach may come at the cost of reduced predictive precision. In the future, more multicenter studies are needed to incorporate more comprehensive assessments including advanced imaging parameters (e.g., LAVi, RV function), detailed medication histories, and lifestyle factors, while employing sophisticated statistical approaches to enhance the model's accuracy and clinical applicability across diverse patient populations.

## 6 Conclusion

This study establishes and internal validates the first clinically CRF prediction models specifically for AF patients after RFCA, utilizing easily available clinical indicators including sex, BMI, 6-minute walk distance, systolic blood pressure, NT-proBNP and glucose.

The models enable rapid outpatient CRF assessment to guide personalized rehabilitation planning and long-term monitoring, while also identifying modifiable risk factors (BMI, blood pressure, and glucose control) for targeted intervention to potentially reduce AF recurrence risk. More importantly, the prediction model will be a practical alternative to CPX testing in resource-limited settings.

## Data Availability

The original contributions presented in the study are included in the article/Supplementary Material, further inquiries can be directed to the corresponding authors.

## References

[1] Joglar JA Chung MK Armbruster AL Benjamin EJ Chyou JY Cronin EM 2023 ACC/AHA/ACCP/HRS guideline for the diagnosis and management of atrial fibrillation: a report of the American College of Cardiology/American Heart Association joint committee on clinical practice guidelines. Circulation. (2024) 149:e1–156. 10.1161/CIR.0000000000001193 38033089 PMC11095842

[2] Lippi G Sanchis-Gomar F Cervellin G. Global epidemiology of atrial fibrillation: an increasing epidemic and public health challenge. Int J Stroke. (2021) 16:217–21. 10.1177/1747493019897870 31955707

[3] Calkins H Hindricks G Cappato R Kim YH Saad EB Aguinaga L 2017 HRS/EHRA/ECAS/APHRS/SOLAECE expert consensus statement on catheter and surgical ablation of atrial fibrillation. Heart Rhythm. (2017) 14:e275–444. 10.1016/j.hrthm.2017.05.012 28506916 PMC6019327

[4] Erhard N Metzner A Fink T. Late arrhythmia recurrence after atrial fibrillation ablation: incidence, mechanisms and clinical implications. Herzschrittmacherther Elektrophysiol. (2022) 33:71–6. 10.1007/s00399-021-00836-6 35006336 PMC8873127

[5] Ko D Chung MK Evans PT Benjamin EJ Helm RH. Atrial fibrillation: a review. JAMA. (2025) 333:329–42. 10.1001/jama.2024.22451 39680399 PMC11774664

[6] Ross R Blair SN Arena R Church TS Després JP Franklin BA Importance of assessing cardiorespiratory fitness in clinical practice: a case for fitness as a clinical vital sign: a scientific statement from the American Heart Association. Circulation. (2016) 134:e653–99. 10.1161/CIR.0000000000000461 27881567

[7] Mujović NM Marinković MM Nedeljković I Marković N Banović M Vučićević V Improvement of maximal exercise performance after catheter-ablation of atrial fibrillation and its prognostic significance for long-term rhythm outcome. J Am Heart Assoc. (2021) 10:e017445. 10.1161/JAHA.120.017445 33506694 PMC7955411

[8] Donnellan E Wazni OM Harb S Kanj M Saliba WI Jaber WA. Higher baseline cardiorespiratory fitness is associated with lower arrhythmia recurrence and death after atrial fibrillation ablation. Heart Rhythm. (2020) 17:1687–93. 10.1016/j.hrthm.2020.05.013 32762978

[9] Pathak RK Elliott A Middeldorp ME Meredith M Mehta AB Mahajan R Impact of CARDIOrespiratory FITness on arrhythmia recurrence in obese individuals with atrial fibrillation: the CARDIO-FIT study. J Am Coll Cardiol. (2015) 66:985–96. 10.1016/j.jacc.2015.06.488 26113406

[10] Guo Y Ng N Hassler S Wu F Miao Jonasson J. Frailty trajectories in Chinese older adults: evidence from the China health and retirement longitudinal study. Innov Aging. (2023) 8:igad131. 10.1093/geroni/igad131 38250747 PMC10798827

[11] Wilkinson C Todd O Clegg A Gale CP Hall M. Management of atrial fibrillation for older people with frailty: a systematic review and meta-analysis. Age Ageing. (2019) 48:196–203. 10.1093/ageing/afy180 30445608 PMC6424377

[12] Ding Y Pan Y Wang M Cao L Xu H Wei L Factors influencing kinesiophobia during the “blanking period” after radiofrequency catheter ablation in patients with atrial fibrillation by the fear-avoidance model. Int J Cardiol. (2022) 363:49–55. 10.1016/j.ijcard.2022.06.021 35716943

[13] Balady GJ Arena R Sietsema K Myers J Coke L Fletcher GF Clinician’s guide to cardiopulmonary exercise testing in adults: a scientific statement from the American Heart Association. Circulation. (2010) 122:191–225. 10.1161/CIR.0b013e3181e52e69 20585013

[14] Šagát P Kalčik Z Bartik P Šiška Ľ Štefan L. A simple equation to estimate maximal oxygen uptake in older adults using the 6 min walk test, sex, age and body mass index. J Clin Med. (2023) 12:4476. 10.3390/jcm12134476 37445511 PMC10342654

[15] Deka P Pozehl BJ Pathak D Williams M Norman JF Alonso WW Predicting maximal oxygen uptake from the 6 min walk test in patients with heart failure. ESC Heart Failure. (2021) 8:47–54. 10.1002/ehf2.13167 33305534 PMC7835615

[16] Peterman JE Arena R Myers J Ades PA Bonikowske AR Harber MP A nonexercise prediction of peak oxygen uptake for patients with cardiovascular disease: DATA FROM THE FITNESS REGISTRY AND THE IMPORTANCE OF EXERCISE INTERNATIONAL DATABASE (FRIEND). J Cardiopulm Rehabil Prev. (2023) 43:115. 10.1097/HCR.0000000000000722 36137212

[17] Giannitsi S Bougiakli M Bechlioulis A Kotsia A Michalis LK Naka KK. 6-minute Walking test: a useful tool in the management of heart failure patients. Ther Adv Cardiovasc Dis. (2019) 13:1753944719870084. 10.1177/1753944719870084 31441375 PMC6710700

[18] Wang Z Li J Jiao K Yan J Zhang T Yu W Effects of 6-week online supervised exercise intervention on patients with different types of coronary heart disease. Acad J Naval Med Univ. (2022) 43:1135–42. 10.16781/j.CN31-2187/R.20211126

[19] Podsiadlo D Richardson S. The timed “up & go”: a test of basic functional mobility for frail elderly persons. J Am Geriatr Soc. (1991) 39:142–8. 10.1111/j.1532-5415.1991.tb01616.x 1991946

[20] Wang Z Yan J Meng S Li J Yu Y Zhang T Reliability and validity of sit-to-stand test protocols in patients with coronary artery disease. Front Cardiovasc Med. (2022) 9:841453. 10.3389/fcvm.2022.841453 36093135 PMC9452740

[21] ATS Committee on Proficiency Standards for Clinical Pulmonary Function Laboratories. ATS Statement: guidelines for the six-minute walk test. Am J Respir Crit Care Med. (2002) 166:111–7. 10.1164/ajrccm.166.1.at1102 12091180

[22] Li J Liu B Wang Z El-Ansary D Adams R Han J Efficacy of a 6-week home-based online supervised exercise program conducted during COVID-19 in patients with post percutaneous coronary intervention: a single-blind randomized controlled trial. Front Cardiovasc Med. (2022) 9:853376. 10.3389/fcvm.2022.853376 35463794 PMC9021490

[23] Buckley BJ Long L Risom SS Lane DA Berg SK Gluud C Exercise-based cardiac rehabilitation for adults with atrial fibrillation. Cochrane Database Syst Rev. (2024) 9:CD011197. 10.1002/14651858.CD011197.pub3 39287086 PMC11406592

[24] Buckley BJ Long L Lane DA Risom S Fitzhugh CJ Berg SK Exercise based cardiac rehabilitation for atrial fibrillation: Cochrane systematic review, meta-analysis, meta-regression and trial sequential analysis. Br J Sports Med. (2025) (in press). 10.1136/bjsports-2024-109149 40730424

[25] Farinatti PT Soares PP. Cardiac output and oxygen uptake relationship during physical effort in men and women over 60 years old. Eur J Appl Physiol. (2009) 107:625–31. 10.1007/s00421-009-1162-y 19711096

[26] Fuchs A Mejdahl MR Kühl JT Stisen ZR Nilsson EJP Køber LV Normal values of left ventricular mass and cardiac chamber volumes assessed by 320-detector computed tomography angiography in the Copenhagen general population study. Eur Heart J Cardiovasc Imaging. (2016) 17:1009–17. 10.1093/ehjci/jev337 26758412

[27] Diaz-Canestro C Pentz B Sehgal A Montero D. Differences in cardiac output and aerobic capacity between sexes are explained by blood volume and oxygen carrying capacity. Front Physiol. (2022) 13:747903. 10.3389/fphys.2022.747903 35370780 PMC8970825

[28] AL-Mhanna SB Batrakoulis A Ghazali WSW Mohamed M Aldayel A Alhussain MH Effects of combined aerobic and resistance training on glycemic control, blood pressure, inflammation, cardiorespiratory fitness and quality of life in patients with type 2 diabetes and overweight/obesity: a systematic review and meta-analysis. PeerJ. (2024) 12:e17525. 10.7717/peerj.17525 38887616 PMC11182026

[29] Lopes S Mesquita-Bastos J Garcia C Bertoquini S Ribau V Teixeira M Effect of exercise training on ambulatory blood pressure among patients with resistant hypertension: a randomized clinical trial. JAMA Cardiology. (2021) 6:1317–23. 10.1001/jamacardio.2021.2735 34347008 PMC8340008

[30] Schröder H Subirana I Elosua R Camps-Vilaró A Tizón-Marcos H Fitó M Measuring cardiorespiratory fitness without exercise testing: the development and validation of a new tool for Spanish adults. J Clin Med. (2024) 13:2210. 10.3390/jcm13082210 38673481 PMC11051378

[31] Simoes MdS Wehrmeister FC Romiti M Gagliardi AdT Arantes RL Dourado VZ. Effect modification of cardiorespiratory fitness, obesity, and physical activity in adults. Int J Sports Med. (2022) 43:561–6. 10.1055/a-1562-6014 34331303

[32] Fu Z Wang Y Wang Y Shi S Li Y Zhang B Linking abnormal fat distribution with HFpEF and diastolic dysfunction: a systematic review, meta-analysis, and meta-regression of observational studies. Lipids Health Dis. (2024) 23:277. 10.1186/s12944-024-02266-y 39217346 PMC11365188

[33] Cho IJ Lee SE Pyun WB. Association of body adiposity with left ventricular concentric remodeling and diastolic dysfunction. Echocardiography. (2024) 41:e15872. 10.1111/echo.15872 38940234

[34] Varvarousis D Kallistratos M Poulimenos L Triantafyllis A Tsinivizov P Giannakopoulos A Cardiac arrhythmias in arterial hypertension. J Clin Hypertens (Greenwich). (2020) 22:1371–8. 10.1111/jch.13989 32772484 PMC8029711

[35] Improta-Caria AC Aras MG Nascimento L De Sousa RAL Aras-Júnior R Souza BdF. MicroRNAs regulating renin–angiotensin–aldosterone system, sympathetic nervous system and left ventricular hypertrophy in systemic arterial hypertension. Biomolecules. (2021) 11:1771. 10.3390/biom11121771 34944415 PMC8698399

[36] Mouzarou A Hadjigeorgiou N Melanarkiti D Plakomyti TE. The role of NT-proBNP levels in the diagnosis of hypertensive heart disease. Diagnostics (Basel). (2025) 15:113. 10.3390/diagnostics15010113 39795641 PMC11719755

[37] de la Cruz-Ares S Cardelo MP Gutiérrez-Mariscal FM Torres-Peña JD García-Rios A Katsiki N Endothelial dysfunction and advanced glycation end products in patients with newly diagnosed versus established diabetes: from the CORDIOPREV study. Nutrients. (2020) 12:238. 10.3390/nu12010238 31963378 PMC7019746

[38] Liu X Gao Y Guo YK Xia CC Shi R Jiang L Cardiac magnetic resonance T1 mapping for evaluating myocardial fibrosis in patients with type 2 diabetes mellitus: correlation with left ventricular longitudinal diastolic dysfunction. Eur Radiol. (2022) 32:7647–56. 10.1007/s00330-022-08800-9 35567605

[39] Wang M Li Y Li S Lv J. Endothelial dysfunction and diabetic cardiomyopathy. Front Endocrinol (Lausanne). (2022) 13:851941. 10.3389/fendo.2022.851941 35464057 PMC9021409

[40] Fudim M Kelly JP Jones AD AbouEzzeddine OF Ambrosy AP Greene SJ Are existing and emerging biomarkers associated with cardiorespiratory fitness in patients with chronic heart failure? Am Heart J. (2020) 220:97–107. 10.1016/j.ahj.2019.11.006 31805424 PMC7008085

[41] Kerr B Brandon L. Atrial fibrillation, thromboembolic risk, and the potential role of the natriuretic peptides, a focus on BNP and NT-proBNP—a narrative review. Int J Cardiol Heart Vasc. (2022) 43:101132. 10.1016/j.ijcha.2022.101132 36246770 PMC9562601

[42] Oeun B Nakatani D Hikoso S Kojima T Dohi T Kitamura T Factors associated with elevated N-terminal pro B-type natriuretic peptide concentrations at the convalescent stage and 1-year outcomes in patients with heart failure with preserved ejection fraction. Circ Rep. (2020) 2:400–8. 10.1253/circrep.CR-20-0051 33693261 PMC7819653

[43] Mueller C McDonald K de Boer RA Maisel A Cleland JGF Kozhuharov N Heart failure association of the European society of cardiology practical guidance on the use of natriuretic peptide concentrations. Eur J Heart Fail. (2019) 21:715–31. 10.1002/ejhf.1494 31222929

[44] Kaminsky LA Arena R Myers J Peterman JE Bonikowske AR Harber MP Updated reference standards for cardiorespiratory fitness measured with cardiopulmonary exercise testing: data from the fitness registry and the importance of exercise national database (FRIEND). Mayo Clin Proc. (2022) 97:285–93. 10.1016/j.mayocp.2021.08.020 34809986

[45] American Thoracic Society, American College of Chest Physicians. ATS/ACCP statement on cardiopulmonary exercise testing. Am J Respir Crit Care Med. (2003) 167:211–77. 10.1164/rccm.167.2.211 12524257

[46] Bumrungkittikul J Thirapatarapong W. Independent predictors and equation of six-minute walk test in post-cardiac surgery. Heart Lung. (2023) 58:134–8. 10.1016/j.hrtlng.2022.12.002 36508845

[47] Ferreira MB Saraiva FA Fonseca T Costa R Marinho A Oliveira JC Clinical associations and prognostic implications of 6-minute walk test in rheumatoid arthritis. Sci Rep. (2022) 12:18672. 10.1038/s41598-022-21547-z 36333405 PMC9636394

[48] Richards AM. The relationship of plasma NT-proBNP to age and outcomes in heart failure. JACC Heart Fail. (2016) 4:746–8. 10.1016/j.jchf.2016.06.006 27522629

[49] Mojón-Álvarez D Giralt T Carreras-Mora J Calvo-Fernández A Izquierdo A Soler C Baseline NT-proBNP levels as a predictor of short-and long-term prognosis in COVID-19 patients: a prospective observational study. BMC Infect Dis. (2024) 24:58. 10.1186/s12879-024-08980-3 38191350 PMC10773093

